# Clinical significance of determining plasma homocysteine: case-control study on arterial and venous thrombotic patients

**DOI:** 10.3325/cmj.2013.54.480

**Published:** 2013-10

**Authors:** Biljana A. Vučković, Velibor S. Čabarkapa, Tatjana A. Ilić, Iva R. Salatić, Zagorka S. Lozanov-Crvenković, Gorana P. Mitić

**Affiliations:** 1 Department of Patophysiology, Faculty of Medicine, University of Novi Sad, Novi Sad, Serbia; 2 Department of Clinical Biochemistry, Centre for Laboratory Medicine, Clinical Centre of Vojvodina, Novi Sad, Serbia; 3 Department of Immunology, Clinic of Nephrology, Clinical Centre of Vojvodina, Novi Sad, Serbia; 4 Institute of Forensic Medicine, Clinical Centre of Vojvodina, Faculty of Medicine, University of Novi Sad, Novi Sad, Serbia; 5 Department of Mathematics and Informatics, Faculty of Sciences, University of Novi Sad, Novi Sad, Serbia

## Abstract

**Aim:**

To determine the differences in plasma homocysteine levels between three MTHFR 677 genotype subgroups in patients with thrombosis and in controls, as well as between patients with thrombosis and controls with the same MTHFR 677 genotype.

**Methods:**

This case-control study was conducted in Clinical Center of Vojvodina, Novi Sad, from June to December 2011. We included 65 patients with either arterial or venous thrombosis (mean age, 40.97 ± 11.38 years) and 65 controls with no history or clinical evidence of any thrombotic event (mean age, 41.23 ± 11.12 years). Patients and controls were age- and sex-matched.

**Results:**

In comparison with controls, thrombotic patients had significantly higher homocysteine levels (12.81 ± 4.94 µmol/L vs 9.82 ± 3.68 µmol/L; *P* < 0.001) and significantly higher incidence of hyperhomocysteinemia (55% vs 22%; *P* < 0.001; odds ratio [OR] = 4.521). There were no significant differences in homocysteine levels between homozygous carriers, heterozygous carriers, and non-carriers of the MTHFR 677 mutation in either thrombotic patients (12.97 ± 5.40 µmol/L vs 12.55 ± 5.71 µmol/L vs 13.27 ± 1.71 µmol/L; *P* = 0.100) or controls (10.07 ± 2.50 µmol/L vs 10.25 ± 4.84 µmol/L vs 9.20 ± 2.44 µmol/L; *P* = 0.651). However, in comparison with controls, homozygous carriers in thrombotic patient group did not have significantly higher levels of homocysteine (12.97 ± 5.40 µmol/L vs 10.07 ± 2.50 µmol/L; *P* = 0.072), but heterozygous carriers (12.55 ± 5.71 µmol/L vs 10.25 ± 4.84 µmol/L; *P* = 0.020) and non-carriers (13.27 ± 1.71 µmol/L vs 9.20 ± 2.44 µmol/L; *P* < 0.001) did. There was no significant difference in homocysteine levels between patients with arterial and venous thrombosis (12.76 ± 3.60 µmol/L vs 12.86 ± 5.51 µmol/L; *P* = 0.990) and between patients with one thrombotic event and those with recurrent thrombotic events (12.14 ± 3.20 µmol/L vs 15.25 ± 8.51 µmol/L; *P* = 0.254).

**Conclusion:**

Plasma homocysteine levels have a greater clinical significance in the prevention of thrombosis and managing its complications than MTHFR 677 genotyping.

Nowadays, thrombosis is a leading cause of morbidity and mortality in most countries (1). The ensuing complications, the most serious of which is myocardial infarction, stroke, and pulmonary thromboembolism may cause long-term and severe disability. They increasingly affect younger populations and generate a great social and economic burden. There are hence continuing efforts to discover biochemical markers that would enable more reliable risk stratification (2). Considering that it is not uncommon that none of the five major risk factors for thrombosis is recognized in a patient presenting with complications of thrombosis, the last few years have witnessed intensified research on new risk factors and unveiling their effects on the pathogenesis of the thrombotic process (3). According to the data for the last ten years reported by the American Heart Association, the most convincing results have been obtained in studies on C-reactive protein (CRP), Lp(a) lipoprotein, apolipoprotein apo(a), fibrinogen, and homocysteine (4).

Homocysteine is an aminothiol compound, which is the main metabolite of an essential amino acid, methionine. Homocysteine metabolism involves either remethylation to methionine or its irreversible metabolism to produce cysteine (5). The so-called homocysteine hypothesis of atherosclerosis, according to which even moderately elevated homocysteine levels may cause progression of atherosclerosis, was first postulated by McCully in 1969 (6), whereas the first evidence on the relation between pathological homocysteine metabolism and coronary disease in general population was provided by Wilcken and Wilcken in 1976 (7). The interest in homocysteine as a risk factor for development of thrombosis has been dramatically increasing since 1990, and it is still in the focus of attention of the scientific community. Hyperhomocysteinemia may result from a number of dietary and lifestyle factors, genetic factors, nutritional deficiencies, and other etiological factors (8-11). The most common form of genetically determined hyperhomocysteinemia is caused by the occurrence of a thermo-liable variant of methylenetetrahydrofolate reductase (tMTHFR), an enzyme involved in homocysteine metabolism, whose enzymatic activity is significantly reduced in hyperhomocysteinemia. The most frequent mutation leading to the manifestation of thermo liability of this enzyme is mutation of the MTHFR 677 gene, caused by alanine to valine substitution (12). The incidence of this mutation is relatively high, although it varies in different ethnic groups (13). It has still not been elucidated whether the degree of hyperhomocysteinemia is significantly higher in homozygous than in heterozygous carriers, however, it has been shown that hyperhomocysteinemia in the presence of this genetic mutation manifests only in the case of low folate levels, making folate deficiency a likely explanation for the expression of the MTHFR thermo liable genotype (14-16). The importance of clear understanding of the role of hyperhomocysteinemia in the etiopathogenesis of thrombosis is underlined by the fact that it can be corrected easily by simple dietary supplementation with group B vitamins and folic acid. If hyperhomocysteinemia is definitely confirmed to be an independent risk factor for thrombosis, this could be an efficient, safe, simple, and cost-effective means of preventing one of the major risk factors for this disease.

Efficient recognition and management of risk factors for thrombosis are very important, and cost-effective methods for detecting risk factors are necessary for routine clinical treatment and prevention of this disease. The aim of our study was to determine the differences in plasma homocysteine levels between three MTHFR 677 genotype subgroups in patients with thrombosis and controls, as well as between patients with thrombosis and controls with same MTHFR 677 genotype.

## Material and methods

### Study design and participants

This case-control study was conducted at the Center for Laboratory Medicine of the Clinical Center of Vojvodina in Novi Sad, Serbia and included 130 participants of both sexes, aged 17 to 59 years. There were 65 thrombotic patients; 36 male and 29 female, aged 17 to 59 years (mean age ± standard deviation 40.97 ± 11.38 years). Among these, 19 had arterial and 46 venous thrombosis. In order to avoid potential influence of acute illness on the studied parameters only patients who had suffered a thrombotic event at least six weeks prior to enrollment were selected. The exclusion criteria were previously verified disorders of the hemostatic mechanism, renal insufficiency, gastrointestinal disorders, autoimmune disease, diabetes, thyroid disorders, malignancy, and consumption of alcohol or substances affecting homocysteine metabolism. At the time of blood sampling, none of the participants had an acute illness that could affect the study results. Blood samples were collected after overnight fasting, with the last meal being a light one. Blood sampling was done with participants sitting. Collected blood was put in a container with ice and centrifuged for half an hour, and analyses were performed immediately thereafter.

The control group consisted of 65 healthy Clinical Center of Vojvodina employees who were personally asked to participate in the study during regular working time; 31 women and 34 men aged 18 to 58 years (mean age ± standard deviation 41.23 ± 11.12 years). Except for the presence of thrombosis, the inclusion and exclusion criteria for the control group were identical to those applied for the cases. Control participants underwent the same laboratory analyses as cases. Prior to the study, informed consent was taken from all the participants. The study protocol was approved by the institutional ethics committee of Clinical Center of Vojvodina.

### Laboratory methods

Plasma homocysteine levels were determined using the fluorescence polarization immunoassay method (17). Hyperhomocysteinemia was defined as plasma homocysteine levels above 12 µmol/L. This cut-off value was selected since the previous upper reference limit of 15 µmol/L has been suggested to be inappropriately high (18). For most European populations, the recommended homocysteine level is less than 10 µmol/L. Homocysteine levels between 10 and 12 µmol/L are considered tolerable, while those ranging from 12 to 15 µmol/L represent a borderline for hyperhomocysteinemia (19). Results of the Framingham study (18) revealed an increase in cardiovascular risk in individuals with homocysteine levels of 11.4 µmol/L, and some authors (19) reported a double increase in the risk of vascular damage associated with homocysteine levels higher than 10.2 µmol/L. Furthermore, it is recommended to determine reference values for every population since there are a variety of factors affecting them (20). Based on the results of healthy Serbian population, the Center of Laboratory Medicine of Clinical Center of Vojvodina adopted 5-12 µmol/L as the reference value. Serum folic acid level was measured using an ion-capture method (21). The assays were performed on an AXSYM instrument with commercially available ABBOTT GmbH &Co.KG (Abbot Diagnostics, Wiesbaden, Germany) immunoassay kits. A polymerase chain reaction (PCR)-based method was employed for MTHFR 677 genotyping. Capillary blood samples were collected from participants and processed shortly afterwards. Genomic DNA was isolated and purified from dried blood spots using the Chelex100®Molecular Grade Resin reagent (Bio-Rad, Hercules, CA, USA) following the manufacturer's instructions. Detection of the MTHFR 677 C or MTHFR 677 T allele in the patients was performed by real-time PCR on ABI PRISM 7000 Sequence Detection System instrument (Applied Biosystems, Foster City, CA, USA) using the assay for allelic discrimination. For the amplification reactions, a set of primers and specific probes for the differentiation between wild allele and MTHFR 677 T alelle, designed and synthesized by the TaqMan SNP Genotyping service (Applied Biosystems, Foster City, CA, USA) was used. Diluted samples and the Taqman Universal PCR Master Mix (Applied Biosystems) set of reagents were added (22). The initial denaturation was carried out for 10- minutes at 95°C. The conditions for 40 cycles of amplification were denaturation at 95°C for 15 seconds and annealing and extension at 60°C for 60 seconds.

### Statistical analysis

Statistical analyses were performed using the Statistica 10 (StatSoft Inc., Tulsa, OK, USA) software. Normality of the distribution was determined using Kolmogorov-Smirnov test. Mean values and standard deviations were calculated for each investigated numerical variable. Comparison of distributions of homocysteine levels for different groups was tested by Mann-Whitney test and Kruskal-Wallis test. Kruskal-Wallis test was followed by multiple comparison of mean rank test if necessary. Comparison of proportions of observed categorical variables was tested using Pearson χ^2^ test. From the contingency tables, odds ratios (OR) for proportions of observed characteristics and corresponding confidence intervals (CI) were calculated and tested for significance. As a measure of correlation, Spearman’s rank correlation coefficient was calculated. The level of significance was set at 0.05.

## Results

Age and homocysteine level were tested for normality of the distributions using Kolmogorov-Smirnov test. The distribution of both variables differed significantly from normal (age *D* = 0.121, *P* < 0.05; homocysteine level, *D* = 0.147, *P* < 0.01). Case and control groups did not differ in age (40.97 ± 11.38 years vs 41.23 ± 11.12 years; *P* = 0.854) and sex (cases: 36 male and 29 female; controls: 34 male and 31 female; *P* = 0.725). We divided the case and control group into three subgroups each, according to age intervals, in order to exclude the possible influence of age on homocysteine level. The first subgroup consisted of participants younger than 31 years, the second of participants aged 31 to 45 years, and the third of participants aged 46 to 59 years. There was no significant difference in sex and age among these subgroups of patients and controls (Table 1).

**Table 1:** Characteristics of patients with thrombosis and the control group

	No. (%) of					
	patients (n = 65)		controls (n = 65)		*P*	
Age (years, mean ± standard deviation)	40.97 ± 11.38		41.23 ± 11.12		0.894*	
Age groups						
<31 (N, mean ± standard deviation)	(14) 24.14 ± 3.90		(11) 25.09 ± 4.32		0.571*	
male		12 (86)		9 (82)		0.792^†^	
female		2 (14)		2 (18)			
31-45 (N, mean ± standard deviation)		(23) 38.35 ± 4.36		(27) 36.18 ± 3.37		0.054*	
male		10 (43)		11 (41)		0.846^†^	
female		13 (57)		16 (59)			
46-59 (N, mean ± standard deviation)		(28) 51.54 ± 3.97		(27) 52.85 ± 3.01		0.173	
male		14 (50)		14 (52)		0.890^†^	
female		14 (50)		13 (48)			
Sex						
male	36 (55.4)		34 (52.3)			0.725^†^
female	29 (44.6)		31 (47.7)			

*Mann-Whitney test.

†χ^2^-test.

Cases had significantly higher mean homocysteine level than controls (12.3 ± 4.94 µmol/L vs 9.2 ± 3.68 µmol/L; *P* < 0.001) and a significantly higher proportion of hyperhomocysteinemia (55% vs 22%; *P* < 0.001; OR 4.522, 95% CI 2.099-9.741). Homocysteine levels were significantly higher in both men with thrombosis than in control men (12.65 ± 5.72 µmol/L vs 9.65 ± 2.35 µmol/L; *P* < 0.001) and women with thrombosis than control women (11.25 ± 3.29 µmol/L vs 8.95 ± 4.77 µmol/L; *P* = 0.024), but there was no difference in the proportion of participants with hyperhomocysteinemia between women with thrombosis and control women (41% vs 23%; *P* = 0.118; OR 2.420, 95% CI 0.789-7.419, *P* = 0.061), while men with thrombosis had significantly higher proportion of hyperhomocysteinemia than control men (55% vs 22%; *P* < 0.001; OR 4.52, 95% CI 2.099-9.741) (Table 2). There was no significant difference in homocysteine levels between men and women in the control group (9.65 ± 2.35 µmol/L vs 8.95 ± 4.77 µmol/L; *P* = 0.396), whereas men with thrombosis had significantly higher homocysteine levels than women with thrombosis (12.65 ± 5.72 µmol/L vs 11.25 ± 3.29 µmol/L; *P* = 0.018). Similarly, control men and women did not significantly differ in the proportion of participants with hyperhomocysteinemia (21% vs 23%; *P* = 0.845; OR 1.125; 95%CI 0.345-3.673, *P* = 0.4226), whereas in the case group it was significantly higher in men (67% vs 41%; *P* = 0.041; OR 2.834, 95%CI 1.029-7.803, *P* = 0.022) (Table 2).

**Table 2:** Comparison of homocysteine levels in male and female participants in case and control groups and the proportion of participants with hyperhomocysteinemia

Characteristics	Case group	Control group	*P* (odds ratio[OR]and 95%confidence interval [CI])
Homocysteine, median (range), µmol/L	12.3 (10.5-14.2)	9.2 (7.7-11.3)	<0.001*
Male	12.65 (11.51-14.85)	9.65 (8.12-11.28)	<0.001*
Female	11.25 (9.01-12.74)	8.95 (7.48-11.12)	0.024*
***P***	0.018*	0.397*	
<31	12.41 (11.33-13.19)	9.80 (8.85-12.04)	
31-45	11.87 (10.46-12.73)	9.20 (6.71-10.76)	
46-59	12.62 (10.4-15.0)	8.92 (7.91-11.8)	
***P***	0.497^†^	0.442^†^	
Proportion of participants with hyperhomocysteinemia, (%)	36 (55)	14 (22)	<0.001^‡^ (4.52, 2.099-9.741)
Male	24 (67)	7 (20)	<0.001^‡^ (7.72, 2.614-22.767)
Female	12 (41)	7 (23)	0.118 ^‡^ (2.42, 0.789-7.419)
***P*** (OR and CI)	0.041 (2.83, 1.029-7.803)	0.845 (1.13, 0.345-3.673)	

*Mann-Whitney test.

†Kruskal-Wallis test.

‡χ^2^-test.

There was no significant difference in homocysteine levels between participants with arterial thrombosis and those with venous thrombosis (12.76 ± 3.60 µmol/L vs 12.87 ± 5.51 µmol/L; *P* = 0.988), and between cases with one thrombotic event and those with recurrent thrombotic events (12.14 ± 3.20 µmol/L vs 15.26 ± 8.51 µmol/L; *P* = 0.254) (Table 3).

**Table 3:** Homocysteine levels in different subgroups of patients.

Subgroups	No. of patients	Homocysteine level in µmol/L (mean±SD*)	*P*
Type of thrombosis:			
Arterial	18	12.76 ± 3.60	0.988^†^
Venous	47	12.87 ± 5.51	
Number of thrombotic events:			
One	51	12.14 ± 3.20	0.254^†^
More than one	14	15.26 ± 8.51	

*SD – standard deviation.

†Mann-Whitney test.

**Figure 1:**
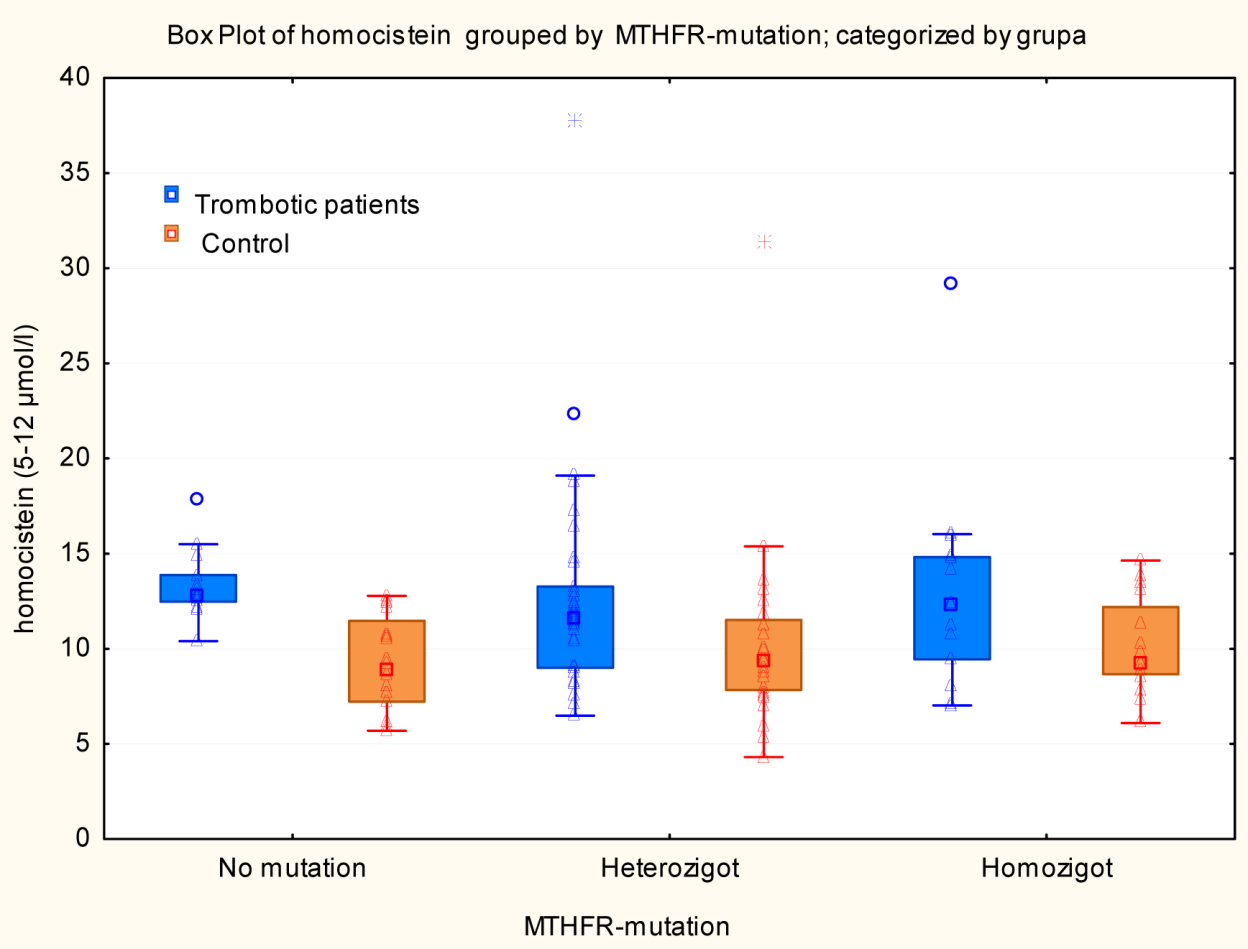
Box Plot of homocysteine levels in different subgroups categorized according to MTHFR genotype

On the basis of the results of genetic testing for mutations in the MTHFR 677 gene, cases and controls were categorized into homozygous carriers of the mutation (677 T/T), heterozygous carriers (677 T/C), and non-carriers of the mutation (677 C/C). There were no significant differences in homocysteine levels between the three case subgroups (12.97 ± 5.39 vs 12.55 ± 5.71 vs 13.27 ± 1.72; *P* = 0.100), as well as between the three control subgroups (10.07 ± 2.50 vs 10.25 ± 4.84 vs 9.21 ± 2.44; *P* = 0.651) (Figure 1). There was also no significant difference in homocysteine levels between 677 T/T carriers in the case group and 677 T/T carriers in control group (12.97 ± 5.39 µmol/L vs 10.07 ± 2.50 µmol/L; *P* = 0.072), but there was a significant difference between 677 T/C carriers in the case group and 677 T/C carriers in the control group (12.55 ± 5.71 µmol/L vs 10.25 ± 4.84 µmol/L; *P* = 0.020), and between non-carriers in the case and non-carriers in the control group (13.27 ± 1.72 µmol/L vs 9.21 ± 2.44 µmol/L; *P* < 0.001) (Figure 1) implying that MTHFR 677 genotype does not have a crucial effect on plasma homocysteine levels.

**Figure 2:**
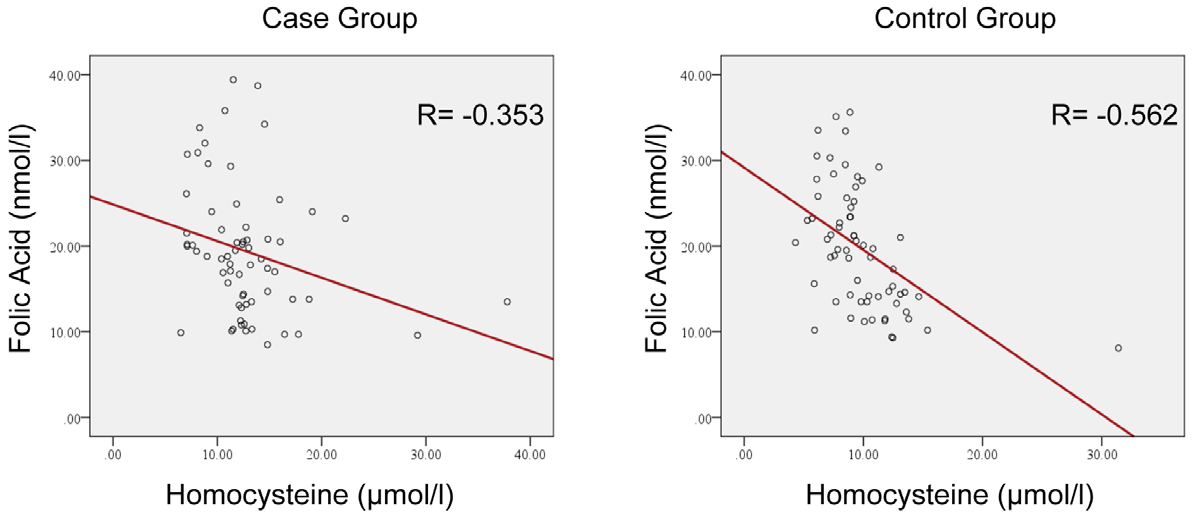
Correlation between homocysteine and folic acid levels in case and control group

In order to investigate whether sex and age influence the results, we analyzed their potential effects. We found no significant association between sex and MTHFR 677 genotype in all participants together (*P* = 0.131), in cases only (*P* = 0.786), and in controls only (*P* = 0.081). Similarly, there was no significant association between age and MTHFR 677 genotype in all participants together (*P* = 0.467), cases only (*P* = 0.451), and controls only (*P* = 0.564) (Table 4). Significant Spearman’s rank correlation coefficients between levels of folic acid and homocysteine were found both in cases and controls (r = -0.353; *P* = 0.004 and r = -0.562; *P* ≤ 0.001, respectively) (Figure 2).

**Table 4:** Frequency of MTHFR genotypes in different subgroups categorized according to age and sex

	No. (%) of participants with				
Participant’s genotype	MTHFR 677CC	MTHFR 677CT	MTHFR 677TT	Total	P*
Cases and controls:					
male	22 (32)	29 (41)	19 (27)	70	0.131
female	13 (22)	35 (58)	12 (20)	60	
total	35	64	31	130	
Cases:					
male	9 (25)	18 (50)	9 (25)	36	0.786
female	6 (21)	17 (59)	6 (20)	29	
total	15	35	15	65	
Controls:					
male	13 (39)	11 (31)	10 (30)	34	0.081
female	7 (23)	18 (58)	6 (19)	31	
total	20	29	16	65	
Cases and controls:					
<31 y	9 (36)	8 (32)	8 (32)	25	0.467
31-45 y	13 (26)	26 (52)	11 (22)	50	
46-59 y	13 (24)	30 (54)	12 (22)	55	
total	35	64	31	130	
Cases:					
<31 y	4 (28)	5 (36)	5 (36)	14	0.451
31-45 y	4 (17)	13 (57)	6 (26)	23	
46-59 y	7 (25)	17 (61)	4 (14)	28	
total	15	35	15	65	
Controls:					
<31 y	5 (46)	3 (27)	3 (27)	11	0.564
31-45 y	9 (33)	13 (48)	5 (19)	27	
46-59 y	6 (23)	13 (46)	8 (31)	27	
total	20	29	16	65	

*χ^2^-test.

In order to elucidate these findings, we first assessed the correlation of homocysteine and folic acid levels and found a negative correlation between these two parameters. After that, we analyzed the levels of folic acid among the subgroups and found that non-carriers of MTHFR 677 mutation on average had significantly lower folate levels than heterozygous and homozygous carriers. Analysis of individual patients showed that the patients with the lowest levels of folic acid in the group of non-carriers of MTHFR 677 mutation had the most pronounced hyperhomocysteinemia.

## Discussion

Consistent with our expectations, this study found that homocysteine levels and the proportion of participants with hyperhomocysteinemia were significantly higher in individuals with thrombosis than in healthy participants. Although a great number of studies shows that elevated homocysteine levels clearly contribute to the development of thrombosis and the ensuing complications (23-25), there are data suggesting that elevated homocysteine levels are merely a consequence and not the cause of vascular disease (26). The proposed pathophysiological mechanisms by which hyperhomocysteinemia disrupts vascular physiology and contributes to the development of thrombosis include endothelial dysfunction as result of oxidation and tyrosine nitration of small and intermediate conductance Ca^2+^-activated potassium channels resulting in impaired endothelium-derived hyperpolarizing factor-mediated relaxation of resistance arterioles (27-29), oxidative modification of low density lipoproteins (LDL) particles (30), increase in lipid uptake and retention by blood vessel walls, increased adhesion of monocytes to the blood vessel wall, stimulation of smooth muscle proliferation (31), activation of inflammatory pathways (32), thrombocytic dysfunction (33), and activation of procoagulant factors of the hemostatic mechanism (27).

According to our results, patients with arterial and venous thrombosis did not have significantly different homocysteine levels, but considering that those subgroups were small and that arterial thrombosis and venous thrombosis patients comparison was not the aim of our study, we are planning on performing a more detailed analysis of these parameters including more participants, alongside with comparison of genotypes in these subgroups. There was no significant difference in homocysteine levels between patients who had one thrombotic event and those with recurrent thrombotic events, however, definite conclusions about this issue require a long-term prospective patient follow-up.

We found no significant differences in homocysteine levels and the proportion of patients with hyperhomocysteinemia between the subgroups of homozygous carriers, heterozygous carriers, and non-carriers of MTHFR 677 among cases. The same results were obtained for control participants. There was also no significant difference between homozygous carriers in thrombotic patients group and homozygous carriers in the control group. However, a significant difference between heterozygous carriers and non-carriers in thrombotic patients group compared to controls was found. These results suggest that the MTHFR 677 genotype does not have a crucial effect on plasma homocysteine levels and that environmental factors may have a greater clinical significance. Other recent studies have shown similar results and according to their findings elevated levels of homocysteine are always significantly higher in the group of patients with documented cardiovascular events, regardless of the genotype. Also, a weak association between the MTHFR C677T polymorphism and cardiovascular disease was established, which suggests that elevated homocysteine levels, rather than the 677 genotype, are associated with development of thrombosis (34,35). Whereas some studies show a clear association between the MTHFR 677 mutation and an increased risk of ischemic heart and brain disease (36), others conclude that the association between the MTHFR 677 mutation and an increased cardiovascular risk manifests only in individuals with low folate levels (37). Therefore, it is important to point out that previous research clearly shows that folate status plays an important role in suppressing negative expression of tMTHFR and in homocysteine concentration (38,39). The influence of folate on homocysteine concentrations is thus one of the best examples of nutritional ecogenetics (7). Furthermore, the finding that homozygosity for the MTHFR 677 mutation requires increased folate intake in order to keep normal homocysteine values corroborates the fact that individual vitamin needs depend on the genetic base (13). However, many other vitamins also have important roles in homocysteine metabolism. Some recent studies show that vitamin B_12_ may have a key role in vitamin therapy used to lower homocysteine levels in stroke prevention interventions. Furthermore, there are data confirming that high consumption of Ω-3 polyunsaturated fatty acids lowers plasma homocysteine (40). Therefore, the best way to evaluate real influence of environmental factors on homocysteine levels is to determine not only the level of folic acid, but also effects of other vitamins and factors that have been proven to influence homocysteine levels, such as smoking or sedentary lifestyle.

Limitations of the study include a small sample, as well as different number of patients with arterial and venous thrombotic events. On the other hand, the goal was not to evaluate the association of the type of thrombosis with homocystein level, but to estimate the significance of genotyping MTHFR mutation. An advantage of the study is that we found a cost-effective approach used for identification of individuals at increased risk for thromboembolic disease.

Finally, we would like to emphasize that hypehomocysteinemia is now a generally recognized risk factor for thrombosis, which is grounded in the results of numerous large-scale clinical and epidemiological studies, as well as basic scientific research on homocysteine metabolism and pathophysiology of elevated homocysteine (36,41-43). On the other hand, it is still unclear whether hyperhomocysteinemia is a causal factor in the occurrence of thrombosis, a consequence of full-fledged disease, or only a biochemical marker of its development. The authors disputing the homocysteine hypothesis postulate that the association between hyperhomocysteinemia and thrombosis is only an indirect one, via factors affecting both homocysteine levels and cardiovascular risk. The latter assumption is supported by the data indicating that the relative risk associated with moderately elevated homocysteine levels has been found to be greater in retrospective than in prospective studies (44-46). Our results suggest that determination of plasma homocysteine concentration has a much more significant clinical role in atherothrombosis risk assessment than MTHFR 677 genotyping. However, definitive conclusions on elevated homocysteine levels as a risk factor for thrombosis can be drawn only after publishing of the results of several long-term studies investigating possibilities of reducing the risk of thrombosis by lowering homocysteine levels with vitamin supplementation therapy (47,48). Until then, the role of homocysteine in etiopathogenesis of thrombosis and particularly the causes of its elevated plasma levels will remain an issue that attracts the attention of scientific community.
